# Are we enabling the next generation to thrive?

**DOI:** 10.1080/17571472.2017.1402496

**Published:** 2017-11-15

**Authors:** Emma McKenzie-Edwards

**Affiliations:** Oxford University (GP Tutor for Hertford College)

Child health and development are clearly top priorities on most parent’s minds, yet parents and non-parents alike we all share concern about these issues. Well managed, they form part of the bedrock of a stable, thriving society. Once we reach adulthood we risk losing connection with what it means to be a child. This sense of disconnection has become even more noticeable with the emergence of the ‘iPad generation’ whose engagement with the world is shaped through technology rather than interpersonal interaction. As part of our initiative to explore these issues, LJPC has previously looked with interest at concepts to do with young minds and their healthy development and how critical connected communities are to their citizens well-being. In LJPC 8.1 Robert Winston and Rebecca Chicot’s paper on children’s mental health and resilience highlighted the importance of early bonding to help develop long-lasting, harmonised, integrated thinking [1]. Susan Hallam, who published her findings from the music for life project in LJPC 8.2, discussed in her work ‘The Power of Music’ the significant positive impact of music on the child’s developing mind [2,3]. We have also looked at how art can influence and enhance learning in Francesco Carelli’s reflections.

Dr. Younie opens this issue with her thoughtful piece ‘Beginners mind’[4], an engaging and fresh look at the ideas around patient engagement focusing on the ideas of an inductive, flow based approach. Flow is an interesting concept. Hungarian psychologist Michaly Csikszentmihalyi described it as a state of heightened focus and immersion in activities such as art, play and work which can allow us to feel fulfilled and happy in our endeavours [5].Children seem to have natural propensity for this beneficial state. Remastering it as an adult is a challenge. LJPC is interested to explore the value of approaches such as mindfulness to enhance flow, mental health and wellbeing. With the worrying trend of increased numbers of child mental health consultations in primary care, Ryan et al. discuss the relative effectiveness of the most widely used parenting programmes on children’s behavioural problems and mental health [6]. The evidence base for the use of programmes in older preschool and school age children is promising. As a number of studies indicate that the first signs of behavioural problems can appear as early as infancy, forthcoming research for programmes targeting the first two years of life may yet encourage us to consider these interventions from an early stage. Should we be exploring attachment and early childcare arrangements more fully in this context?

Xiajuan Tan invites us to consider the efficacy of intensive home-based intervention, Multisytematic Therapy, when managing young people with severe antisocial behaviour and emotional disorders [7]. Significant, positive effects on delinquency, incarceration and suicide attempts are discussed in the systematic review. Antisocial behaviour and conduct disorders are the most common behavioural and mental health problems in children and young people globally and so warrant serious consideration. Knowledge that community intervention not only appears to be cost effective but more efficacious compels us to step up to the plate. We delve further into the community aspect of child health with Dr. Samantha Ross’s paper on Preschool growth and nutrition services [8]. Ross discuses practical management in the community of a growing clinical problem. The message of creating a coherent ‘joined-up’ approach to tackling childhood obesity is one that translates between the community and the hospital setting and encourages us to reimagine how care should be delivered. Francesco Carelli rounds off the issue with his insights into teaching and learning through the art of Kasimir Malevich [9]. Carelli and his grandson consider Malevich’s work together and encourage us to ‘look under the surface’, to think about more than just what we see, to develop critical, independent thought. He demonstrates that we can thoughtfully stimulate a young mind. A beginners mind in action.

Emerging from the work in this issue is the idea that children are facing some tough challenges in terms of mental and physical health. Our communities become enriched by happy, well adjusted, confident young people. We need to consider significant investment in their care and development a priority. Our attendance to children’s emotional and physical wellbeing in the primary care setting is particularly important as it is in this environment that children seem to glean a great deal from intervention. It also seems we have something to learn from our children if we want to achieve a happier state of being.

How can we continue to move forward? LJPC wants to continue to explore this territory looking for fresh concepts and ideas. Our challenge, as parents, health care professionals and citizens is to consider how we can continue to enable the next generation to thrive, rather than just survive.
